# Supplementary material for: Evaluation of the discriminatory potential of antibodies created from synthetic peptides derived from wheat, barley, rye and oat gluten
Source: PLoS One. 2021 Sep 23;16(9):e0257466. doi: 10.1371/journal.pone.0257466 (PMC8459967; doi:10.1371/journal.pone.0257466)
Supplement: S1 Table — (DOCX) [file pone.0257466.s001.docx]

S1 Table. Consensus sequences obtained from multiple alignments sequences (MSA) and compilation

|  | 1∙∙∙∙∙∙∙∙∙∙12∙∙∙∙∙∙∙∙∙∙24∙∙∙∙∙∙∙∙∙∙36∙∙∙∙∙∙∙∙∙∙48∙∙∙∙∙∙∙∙∙∙60∙∙∙∙∙∙∙∙∙72 |
| --- | --- |
|  | \|∙∙∙∙\|∙∙∙∙∙\|∙∙∙∙∙\|∙∙∙∙∙\|∙∙∙∙∙\|∙∙∙∙∙\|∙∙∙∙∙\|∙∙∙∙∙\|∙∙∙∙∙\|∙∙∙∙∙\|∙∙∙∙∙\|∙∙∙∙∙\| |
| >Consensus/α-gliadin/1-72 | MKTFLILALLAIVATTATTAVRVPVPQQVEWPQQQPLLQPQNPSQQQPQEQVPLVQQQQFLGQQQQQFPGQQ |
| >Consensus/γ-gliadin/1-72 | TIMKTLLILTILAMATTIATANMQVDPSGQVQWPQQQQPFPQPQQPFSQQPQQTFPQPQPQQTFPHQPQQQF |
| >Consensus/ω-gliadin/1-72 | MKTFIIFVLLAMAMNIASAASRQPVPQLSPRGKELQTPQEQFPQQQQFPFPPQQFPQQQIPQQHQPYPQQPQ |
| >Consensus/LMW-GS/1-72 | YQQQQPIQQQMKTFLVFALLAVAATSAIAQMETSCIPGLERQQFPRPQQFPQQQILQQHQIPWQQQPLPPQQ |
| >Consensus/HMW-GS/1-72 | MAKRLVLFAAVVVALVALTAAEGEASGQLQCERELQELQESSLEACRQVVDQQLAGRLPWSTGLQMRCCQQL |
| >Consensus/B-hordein/1-72 | MKTFLIFALLAIAATSTIAQQQPFPQQPFPQQPQPYPPQQQPQPYPQQPFQPQQPFPQQTIPQQPQPYPQQP |
| >Consensus/B1-hordein/1-72 | MKTFLIFALLAIAATSTIAQQQPFPQQPIPQQPQPFPQQPQPYPQQPFPPQQPFPQQPVPQQPQPYPQQPQP |
| >Consensus/B3-hordein/1-72 | MKTFLIFALLAIVATSTIAQQQPYPQQPQPFPQQPIPQQPQQFPQQPQPYPQQPQPFPQQPIPQQPQPYPQQ |
| >Consensus/C-hordein/1-72 | PISRSGSARRKNKPRKLEIVSDEDILMMKTFLTFVELLLAMAMSIVTTELPSEIARQLQEILQNPSHQELQS |
| >Consensus/D-hordein/1-72 | MAKRLVLFVAVIVALVALTTAEREINGNNIFLDSRSRQLQCERELQESSLEACRRVVDQQLVGQLPWSTGLQ |
| >Consensus/γ1-hordein/1-72 | MKILIILTILAMATTFATSEMQVNPSVQVQPTQQQPYPESQQPFISQSQQQFPQPQQPFPQQPQQPFPQSQQ |
| >Consensus/γ3-hordein/1-72 | MSFVYLFPFEIVALDMNLMAMKIFLLFALLGLATTITTATVQFDPSSGHGLIVQRPQQSYPQWQPVPQQQPF |
| >Consensus/γ-secalin/1-72 | MKTLLMLAILAMATTIATANMQVNPSGQVQCPQQQPFPQPQQSSPQQPQQPFPQQSQQPFPQQPQQSSPQPQ |
| >Consensus/ω-secalin/1-72 | MKTFLIFVLAMTMSIITTARQLNPSEQELQSPQQPVPKEQSYPQQPYPSHQPFPTPQQYSPYQPQQPFPQPQ |
| >Consensus/avenin/1-72 | MKTFLILALLAMAAAVATATTMATAQYDPSEQYQPYPEQQQPFLQQQQPLQQQQQPFMQQQQQPFVQQQQPF |
|  |  |
|  | 73∙∙∙∙∙∙∙∙∙84∙∙∙∙∙∙∙∙∙∙96∙∙∙∙∙∙∙∙∙∙108∙∙∙∙∙∙∙∙∙120∙∙∙∙∙∙∙∙∙132∙∙∙∙∙∙∙144 |
|  | \|∙∙∙∙\|∙∙∙∙∙\|∙∙∙∙∙\|∙∙∙∙∙\|∙∙∙∙∙\|∙∙∙∙∙\|∙∙∙∙∙\|∙∙∙∙∙\|∙∙∙∙∙\|∙∙∙∙∙\|∙∙∙∙∙\|∙∙∙∙∙\| |
| >Consensus/α-gliadin/73-144 | QPFPHQPQQPYPQPQPFPSQQPYLQLPQAQQPLQPFPQPQLPYPPQPQLPYPLQPQLPYPQPQQPQQPQPQP |
| >Consensus/γ-gliadin/73-144 | PQQQQQPFPQPQQPQQPFLQPQQPQQTFPHQPQQQFPQPQQQPYPQQPQQPFPQQPQQPVPQQPQQQFPQQP |
| >Consensus/ω-gliadin/73-144 | PYPQQQPYPSQQPFPTPQQQFPQQSQQPFQIPQQPQPQPQQPTPLQPQQPFPQQQQQPFPQQPQQPQQPFPQ |
| >Consensus/LMW-GS/73-144 | TLPQQQPPFSQQQQQQQPIQQQPIQQQQQPFPQQPPFSQQQQQPPLSQQQQPPFSQQQQPPFSQQQQPPLSQ |
| >Consensus/HMW-GS/73-144 | RDVSPKCRPVAVSPVARQYEQQPVVPPKGGSFYPGETTPPQQLQQMIFWGIPAQLLRRYYPSVTSPQQGSYY |
| >Consensus/B-hordein/73-144 | QPYPQQPFPPQQQPYPQQPQPFPQQPPPFWPQQPFPQQPPFGLQQPILSQQQPCTPQQTPLPQGQLYQTLLQ |
| >Consensus/B1-hordein/73-144 | YPQQPFPPQQPFPQQPPFWPQQPFPQQPPFGLQQPILSQQQPCTPQQTPLPQGQLYQTLLQLQIPYVHPSIL |
| >Consensus/B3-hordein/73-144 | PQPFPQQPIPQQPQPYPQQPQPFPLQPFPSQQPFPQQPPFWQQQPVLSQQQPCTQEQTPLLQEQQDQMLLQV |
| >Consensus/C-hordein/73-144 | PQQPFLKQQSYLQQPYPQNPYLPQQPFPVQQPFPTPQQFFPYLPQQTFPQSQQPTPLQPQQPFPLQPQPPQQ |
| >Consensus/D-hordein/73-144 | MQCCQQLRDVSPECRPVALSQVVRQYEQQTEVPSKGGSFYPGGTAPPLQQGGWWGTSVKWYYPDQTSSQQSW |
| >Consensus/γ1-hordein/73-144 | QCLQQPQHQFPQPTQQFPQRPLLPFTHPFLTFPDQLLPQPPHQSFPQPPQSYPQPPLQPFPQPPQQKYPEQP |
| >Consensus/γ3-hordein/73-144 | PQQDPQQPYPQQQLLPQQQPFSQQQQLPQQHPFPQQMPQQQFPQQMPLQPQQQFPQQMPLLPQQPPQFPQQQ |
| >Consensus/γ-secalin/73-144 | QPYPQQPFPQQPQQPFPQQPQQPFPQQPQQPYPQQPQQPFPQQPQQPVPQQPQQPFPQQPQQPFPQQPQQPV |
| >Consensus/ω-secalin/73-144 | QPTPIQPQQPFPQQPQQPFPQQPQQPFPQQPQQPFPQQPQQQLPLQPQQPFPQPQQPIPQQPQQPIPQQPQQ |
| >Consensus/avenin/73-144 | VQQQQPFVQQQPFVQQQQQQPFMQQQQPFLQQQQQQFLQQLLQQQLNPCRQFLVQQCSPVAMVPFLRSQILR |
|  |  |
|  | 145∙∙∙∙∙∙∙∙156∙∙∙∙∙∙∙∙∙168∙∙∙∙∙∙∙∙∙180∙∙∙∙∙∙∙∙∙192∙∙∙∙∙∙∙∙∙204∙∙∙∙∙∙∙216 |
|  | \|∙∙∙∙\|∙∙∙∙∙\|∙∙∙∙∙\|∙∙∙∙∙\|∙∙∙∙∙\|∙∙∙∙∙\|∙∙∙∙∙\|∙∙∙∙∙\|∙∙∙∙∙\|∙∙∙∙∙\|∙∙∙∙∙\|∙∙∙∙∙\| |
| >Consensus/α-gliadin/145-216 | FRPQQPYPQPQPQYSQPQQPISQQQQQQQQQQQQQQQQQQQQQQQQQQQQQQQQQQQQQQQQQQQQILQQIL |
| >Consensus/γ-gliadin/145-216 | QQPVPQQPQQPFPQQPQQPFPQTQQQPVPQQPLQQFPQQPQQPFPQQPQQPFPQPQQQQFPQPQQPQQPFPQ |
| >Consensus/ω-gliadin/145-216 | QQFPQQKLPQQQEFPQQQISQPQQPLPQQQQIPQQPQQFPWQPQQPFPQTQQQQSFPLQQFQPQQPFPQQPQ |
| >Consensus/LMW-GS/145-216 | PQQPFPQQPQQQQPPFSQQQFPQQQLPQQEFSQQQQQPILPQQPPFSQQQQPPFSQQQQQIPQQPQQFLQQQ |
| >Consensus/HMW-GS/145-216 | PGQASPQQPGQGQQPGQGQQPGQGQQDQQPEQGQQSGQGQQGYYPTSPGQPQQPGQWQQPGQGQQGYYPTSP |
| >Consensus/B-hordein/145-216 | LQIPFVHPSILQQLNPCKVFLQQQCSPVRMPQRIARSQMLQQSSCHVLQQQCCQQLPQIPEQFRHEAIRAIV |
| >Consensus/B1-hordein/145-216 | QQLNPCKVFLQQQCSPVRMPQLIARLQMLQLSSCHVLQQQCCQQLPQISEQFRHEAIRAIVYSIFLQEQPQQ |
| >Consensus/B3-hordein/145-216 | QIPFVHPSILQQLNPCKVFLQQQCSPVAMSQRIARSQMLQQSSCHVLQQQCCQQLPQIPEQLRHEAVRAIVY |
| >Consensus/C-hordein/145-216 | PFPQPQQPFPWQPQQPFPQPQQPIPQQPQQPFNQQPQQIIPQQPQQPFPQQPQQPFPQPQQPFPWQPQQPFL |
| >Consensus/D-hordein/145-216 | QGQQGYHQSVTSSQQPGQGQQGSYPGSTFPQQPGQGQQPGQRQPWSYPSATFPQQPGQGQGQQGYYPGATSL |
| >Consensus/γ1-hordein/145-216 | QQPFPWQQPTIQLYLQQQLNPCKEFLLQQCRPVSLLSYIWSKIVQQSSCRVMQQQCCLQLAQIPEQYKCTAI |
| >Consensus/γ3-hordein/145-216 | PFAQPQQPLTQQPYPQEQPLSQQQPSVEEQQQLNVCKEFLLQQCNPNEKVSSLQSVIPFLRPQTWQQNSCQL |
| >Consensus/γ-secalin/145-216 | PQQPQQSSPQPQQPFPQQPFPQQPQQPVPQQPQQQFPQQPQQSFPQQPQQPVPQQPLQQFPQQPQQPFPQQP |
| >Consensus/ω-secalin/145-216 | SFPQQPQRPEQQFPQQPQQIIPQQTQQPFPLQPQQPFPQQPQRPFAQQPEQIISQQPFPLQPQQPFSQPQQP |
| >Consensus/avenin/145-216 | QSSCQVMRQQCCRQLAQIPEQLRCPAIHSVVQAIIMQQQQQQQQQPQLQQQFVQPQLQQQLFQPQLQIAQQF |
|  |  |
|  | 217∙∙∙∙∙∙∙∙228∙∙∙∙∙∙∙∙∙240∙∙∙∙∙∙∙∙∙252∙∙∙∙∙∙∙∙∙264∙∙∙∙∙∙∙∙∙276∙∙∙∙∙∙∙288 |
|  | \|∙∙∙∙\|∙∙∙∙∙\|∙∙∙∙∙\|∙∙∙∙∙\|∙∙∙∙∙\|∙∙∙∙∙\|∙∙∙∙∙\|∙∙∙∙∙\|∙∙∙∙∙\|∙∙∙∙∙\|∙∙∙∙∙\|∙∙∙∙∙\| |
| >Consensus/α-gliadin/217-288 | LQQQLIPCRDVVVLQQHNIALVHHGSSQVLQQSTYQLLQQLCCQQLWQIPEQSRCQAIHNVVHAIILHQQQK |
| >Consensus/γ-gliadin/217-288 | PQQPQQLFPQTQQSSPQQPQQVTQQPQQPFPQAQPPQQSSPQSQQPFPQQPQQPFPQPQTQQSIQQPQQPFP |
| >Consensus/ω-gliadin/217-288 | QPQQPQQFPQQQQIPPFPQPQLPFPQQPQQIIPQQQQPQQPFPQQPFPLQPQQPFPQQPQFPQQPFPQQPQQ |
| >Consensus/LMW-GS/217-288 | PQPPFSQQQQPVLPQQPQQQLPQQQQIPQQSFIPQQPQQPIPQQQQSQQQPKPFPQQQPQFPQQQFPFSQQQ |
| >Consensus/HMW-GS/217-288 | QQPGQGQQGYYPSSLQQPGQGQQGHYPGQGQQSGQGQQGYYPTSPQQPGQGQQPGQGQQRQQPGQGQQLRQG |
| >Consensus/B-hordein/217-288 | YSIFLQEQPQEDFVQQQQQLQQSVQGVSQPQQAIQQLQQEQVGQCSFQQPQPQQLGQQPQQQQVPQSVFLQP |
| >Consensus/B1-hordein/217-288 | SVQGVSQTQQQLQQEKVGQCSFQQPQPQQLGQPQQVPQSVFLQPHQIAQLEATTSIALRTLPRMCNVNVPLY |
| >Consensus/B3-hordein/217-288 | SIVLQEQSLQLVQGVSQPQQQSQQQQVGQCSFQQPQPQQGQQQQVPQSVFLQPHQIAQLEATTSIALRTLPT |
| >Consensus/C-hordein/217-288 | QPLQLFPLQPQQPFPWQPQQPFPQPQQPIAHQPQQPFSEQPQQSFSQQPQQPFPLQPQQPFPQQPQQPFPQQ |
| >Consensus/D-hordein/217-288 | LQPGQGQQGPYQSATSPQQPGQGQGQQETYPIATSPHQPGQWQQPGQGQQGYYPSVTSPQQSGQGQQGYPST |
| >Consensus/γ1-hordein/217-288 | DSIVHAIFMQQGQRQGVQIVQQQPQPQQVGQCVLVQGQGVVQPQQLAQMEAIRTLVLQSVPSMCNFNVPPNC |
| >Consensus/γ3-hordein/217-288 | KRQQCCRQLANINEQSRCPAIQTIVHAIVMQQQQQQVQQQVGHGFIQSQPQQLGQGMPIYPQQQPGHGFFLP |
| >Consensus/γ-secalin/217-288 | QQPVPQQSQQPVPQQSQQPFPQTQQPQQPFPQPQQPQQLFPQTQQQSSPQQPQQVTSQPQQPFPQAQPPQQS |
| >Consensus/ω-secalin/217-288 | FPQQPGQIIPQQPQQPSPLQPQQPFSQQPQRPQQPFPQQPQQIIPQQPQQPFPLQPQQPVPQQPQRPFGQQP |
| >Consensus/avenin/217-288 | PTQSTWALSAFQPQLQQVTIAGKSSLQAQLQQQLLANQLQLQQGIFQPQTQQQLLQQQLQQQVFQPQLQQQV |
|  |  |
|  | 289∙∙∙∙∙∙∙∙300∙∙∙∙∙∙∙∙∙312∙∙∙∙∙∙∙∙∙324∙∙∙∙∙∙∙∙∙336∙∙∙∙∙∙∙∙∙348∙∙∙∙∙∙∙360 |
|  | \|∙∙∙∙\|∙∙∙∙∙\|∙∙∙∙∙\|∙∙∙∙∙\|∙∙∙∙∙\|∙∙∙∙∙\|∙∙∙∙∙\|∙∙∙∙∙\|∙∙∙∙∙\|∙∙∙∙∙\|∙∙∙∙∙\|∙∙∙∙∙\| |
| >Consensus/α-gliadin/289-360 | QQQQQQQQLQQQQQQQQQQQQQQQQQQQQQQQQQQQQQQQPSSQVSFQQPQQQYPSGQGSFQPSQQNPQAQG |
| >Consensus/γ-gliadin/289-360 | QSQQPQQPFPQSQPQTQIIQQSIPQPQQPFPQYQQAWEPQQPISPPQQPFPQQPQQQFPQPQQPQQSFPQQQ |
| >Consensus/ω-gliadin/289-360 | IIPQQQQIPQQPQQFPQQQQFPQQQQPQQQQFPQPQQIFPELSQQPFPQQPQQPFPLQPQQPFSQPQQPFPQ |
| >Consensus/LMW-GS/289-360 | QPPFSQQQQPPHQQIAQQPQFSQQQQPIQQQQPVLPQQPPFSQQQQPPFSQQQQQPCPLFPQPPFSQQQQQP |
| >Consensus/HMW-GS/289-360 | QQGQQIGQGQPGYYPTSPQQPGQRQQPGQGQQIGQLQQPAQGQQGQQPEQGQQGQQPGQGQQLGQGQQGQQP |
| >Consensus/B-hordein/289-333 | HQIAQLEATTSIALRTLPTMCNVNVPLYDITTSMPFGVGTRVGVY--------------------------- |
| >Consensus/B1-hordein/289-301 | DIMPPDFWHRVGV----------------------------------------------------------- |
| >Consensus/B3-hordein/289-310 | MCSVNVPLYRIVPLAIDTRVGV-------------------------------------------------- |
| >Consensus/C-hordein/289-360 | PQQIIFQQPQQSYPVQPQQPFPQPQPVPQQRPQQASPLQPQQPFPQGSEQIIPQQPFPLQPQQPFPQQPQPF |
| >Consensus/D-hordein/289-360 | TSPQQSGQGQQLGQGQQPGQGQQGYPSATFPQQPGQWQQGSYPSTTSPQQSGQGQQGYNPSGTSTQQPGQVQ |
| >Consensus/γ1-hordein/289-305 | STIKAPFVGVVTGVGGQ------------------------------------------------------- |
| >Consensus/γ3-hordein/289-336 | QQQAQQFNLVRSLVIQTLPMLCNVHVPPYCSTTTAPFGSMPTGIGGQK------------------------ |
| >Consensus/γ-secalin/289-360 | SPQSQQPYPQEPQQLFPQSQQQPVPQQPQQPFPQPQQPQQPFPQPQPQPVPQQQTQQSIPQPQQPFPQPQQP |
| >Consensus/ω-secalin/289-360 | EQIISQRPQQPFPLQPQQPFSQPQQPFPQQPGQIIPQQPQQPFPLQPQQPFPQQPEQIISQQPQQPFPLQPQ |
| >Consensus/avenin/289-354 | FQPQLQQVFIPPQTQGFFQPQQQAQFEGMRAFALQALPAMCDVYVPPHCPVATVPLGGIAAGIGGC------ |
|  |  |
|  | 361∙∙∙∙∙∙∙∙372∙∙∙∙∙∙∙∙∙384∙∙∙∙∙∙∙∙∙396∙∙∙∙∙∙∙∙∙408∙∙∙∙∙∙∙∙∙420∙∙∙∙∙∙∙432 |
|  | \|∙∙∙∙\|∙∙∙∙∙\|∙∙∙∙∙\|∙∙∙∙∙\|∙∙∙∙∙\|∙∙∙∙∙\|∙∙∙∙∙\|∙∙∙∙∙\|∙∙∙∙∙\|∙∙∙∙∙\|∙∙∙∙∙\|∙∙∙∙∙\| |
| >Consensus/α-gliadin/361-432 | SVQPQQLPQFEEIRNLALQTLPAMCNVYIPPYCTTMNFANVLPTFMSVGFEIPSSLTIAPFGIFGTNYRELI |
| >Consensus/γ-gliadin/361-432 | QPLIQPSLQQQLNPCKNFLLQQCNPVSLLSLLQSPVSSLWSMILPRSDCQVMQQQCCQQLAQIPQQLQCAAI |
| >Consensus/ω-gliadin/361-432 | QPGQIIPQQPQQPSPLQPQQPFPQQQQRPQQPFPQQPQQIIPQQPQQPFPLQPQQPFPQPQQRPFPQQPQQP |
| >Consensus/LMW-GS/361-432 | QPVLPQQPPFSQQQQPPFPQQPPFFQQQQPILPQQPPFSQQQQQPQQPFPHQQLPQQQIPVVQPSILQQLNP |
| >Consensus/HMW-GS/361-432 | GQKQQPGQGQQGQQSGQGQQGYYPTSPQQLGQGQQPGQWQQSGQGQLGYYPTSPQQSGQGQQPGQGQSGYYP |
| >Consensus/B-hordein/334-333 | ------------------------------------------------------------------------ |
| >Consensus/B1-hordein/302-301 | ------------------------------------------------------------------------ |
| >Consensus/B3-hordein/311-310 | ------------------------------------------------------------------------ |
| >Consensus/C-hordein/361-412 | SQQPLPQPQQPFRQQAELIIPQQPQQPFPLQPHQPHQPYTQQTIWSMVALLG-------------------- |
| >Consensus/D-hordein/361-432 | QLGQGQQGYYPIATSPQQPGQGQQLGQGQQPGHGQQLVQGQQQGQGQQGHYPSMTSPHQTGQGQKGYYPSAI |
| >Consensus/γ1-hordein/306-305 | ------------------------------------------------------------------------ |
| >Consensus/γ3-hordein/337-336 | ------------------------------------------------------------------------ |
| >Consensus/γ-secalin/361-432 | LVFPQSQEPFPQVHQPQQPSPQQQQPSIQLSLQQQLNPCKNVLLQQCSPVALVSSLRSKIFPQSECQVMQQQ |
| >Consensus/ω-secalin/361-393 | QPSPQQPQLPFPQPQQPFVVVVIGIGGQSNKEI--------------------------------------- |
| >Consensus/avenin/355-354 | ------------------------------------------------------------------------ |
|  |  |
|  | 433∙∙∙∙∙∙∙∙444∙∙∙∙∙∙∙∙∙456∙∙∙∙∙∙∙∙∙468∙∙∙∙∙∙∙∙∙480∙∙∙∙∙∙∙∙∙492∙∙∙∙∙∙∙504 |
|  | \|∙∙∙∙\|∙∙∙∙∙\|∙∙∙∙∙\|∙∙∙∙∙\|∙∙∙∙∙\|∙∙∙∙∙\|∙∙∙∙∙\|∙∙∙∙∙\|∙∙∙∙∙\|∙∙∙∙∙\|∙∙∙∙∙\|∙∙∙∙∙\| |
| >Consensus/α-gliadin/433-504 | MSPGGTTSFLDVLRGGARGLLDGSLSQRHGYYYGGPAIGSGNGMLMTPPAVSFGIPVPMQQHGDLVVGGNGI |
| >Consensus/γ-gliadin/433-504 | HSVVHSIIMQQEQQEQQQIQPQQQQQQQSGQGVQSQPQQQQQQQGVQILRPLSQQQQVGQQQPQQPQGQLVQ |
| >Consensus/ω-gliadin/433-504 | FPQQQQQSQQQFPQPQQQFPQQQQPFPQQQFPQQQQFPQQQQPQQPFPLQPQQPFPQQPGQIFPQQPNQQPF |
| >Consensus/LMW-GS/433-504 | CKVFLQQQCSPVAMPQRLARSQMLQQSSCHVMQQQCCQQLPQIPEQSRYEAIRAIIYSIILQEQQQVQGFVQ |
| >Consensus/HMW-GS/433-504 | TSPQQPGQGQQPGQPGQLQQPGQGQQSGQGQQGQQPGQGQQGQQPGQGQQPGQGQPGYYPTSPQQSGQGQPG |
| >Consensus/B-hordein/334-333 | ------------------------------------------------------------------------ |
| >Consensus/B1-hordein/302-301 | ------------------------------------------------------------------------ |
| >Consensus/B3-hordein/311-310 | ------------------------------------------------------------------------ |
| >Consensus/C-hordein/413-412 | ------------------------------------------------------------------------ |
| >Consensus/D-hordein/433-504 | SPQQSGQGQQGYQPSGASSQGSVQGACQHSTSSPQQQAQGCQASSPKQGLGSLYYPSGAYTQQKPGQGYNPG |
| >Consensus/γ1-hordein/306-305 | ------------------------------------------------------------------------ |
| >Consensus/γ3-hordein/337-336 | ------------------------------------------------------------------------ |
| >Consensus/γ-secalin/433-504 | CCQQLAQIPQQLQCAAIHSVVHAIIMQQEQREQQGVQILLPQSHQQHVGQGALAQVQGIIQPQQLSQLEVVR |
| >Consensus/ω-secalin/394-393 | ------------------------------------------------------------------------ |
| >Consensus/avenin/355-354 | ------------------------------------------------------------------------ |
|  |  |
|  | 505∙∙∙∙∙∙∙∙516∙∙∙∙∙∙∙∙∙528∙∙∙∙∙∙∙∙∙540∙∙∙∙∙∙∙∙∙552∙∙∙∙∙∙∙∙∙564∙∙∙∙∙∙∙576 |
|  | \|∙∙∙∙\|∙∙∙∙∙\|∙∙∙∙∙\|∙∙∙∙∙\|∙∙∙∙∙\|∙∙∙∙∙\|∙∙∙∙∙\|∙∙∙∙∙\|∙∙∙∙∙\|∙∙∙∙∙\|∙∙∙∙∙\|∙∙∙∙∙\| |
| >Consensus/α-gliadin/505-576 | GAATASIFQGATSEEGDDGMGGVMGLQWQPQVGNGGGGGGVSGGVHHLGTGNNVTMGNSNIHNNNNNDSGGD |
| >Consensus/γ-gliadin/505-562 | GQGIIQPQQPAQLEVIRSLVLQTLPTMCNVYVPPDCSTINAPFASIVAGASIGGQYRA-------------- |
| >Consensus/ω-gliadin/505-576 | PQQPQQPFPQQQQQPQQQFPQQQFPQQPQQPFQFPQQQQFPQQQQLTQQQFPQPQQSPEQQPSILQPQQPLP |
| >Consensus/LMW-GS/505-576 | PQQQQPQQSQGSIQPQQQQPQQLGQGVSQPQQQSQQQQQLGQCSFQQPQQLQQQLGQQPQQQQLGQQPQQQQ |
| >Consensus/HMW-GS/505-576 | YYPTSSQQPTQSQQPGQGQQGQQVGQGQQAQQPGQGQQLGQGQPGYYPTSPLQSGQGQSGQQPGQGQQPGQG |
| >Consensus/B-hordein/334-333 | ------------------------------------------------------------------------ |
| >Consensus/B1-hordein/302-301 | ------------------------------------------------------------------------ |
| >Consensus/B3-hordein/311-310 | ------------------------------------------------------------------------ |
| >Consensus/C-hordein/413-412 | ------------------------------------------------------------------------ |
| >Consensus/D-hordein/505-576 | GTSPLHQQGGGFGGGLTTEQPQGGKQPFHCQQTTVSPHQGQQTTVSPHQGQQTTVSPHQGQQTTVSPHQGQQ |
| >Consensus/γ1-hordein/306-305 | ------------------------------------------------------------------------ |
| >Consensus/γ3-hordein/337-336 | ------------------------------------------------------------------------ |
| >Consensus/γ-secalin/505-540 | SLVLQNLPTMCNVYVPRQCSTIQAPFASIVTGIVGH------------------------------------ |
| >Consensus/ω-secalin/394-393 | ------------------------------------------------------------------------ |
| >Consensus/avenin/355-354 | ------------------------------------------------------------------------ |
|  |  |
|  | 577∙∙∙∙∙∙∙∙588∙∙∙∙∙∙∙∙∙600∙∙∙∙∙∙∙∙∙612∙∙∙∙∙∙∙∙∙624∙∙∙∙∙∙∙∙∙636∙∙∙∙∙∙∙648 |
|  | \|∙∙∙∙\|∙∙∙∙∙\|∙∙∙∙∙\|∙∙∙∙∙\|∙∙∙∙∙\|∙∙∙∙∙\|∙∙∙∙∙\|∙∙∙∙∙\|∙∙∙∙∙\|∙∙∙∙∙\|∙∙∙∙∙\|∙∙∙∙∙\| |
| >Consensus/α-gliadin/577-607 | DNNGGSSRDCYWINNGGSNPWQSLLNSSSLM----------------------------------------- |
| >Consensus/γ-gliadin/563-562 | ------------------------------------------------------------------------ |
| >Consensus/ω-gliadin/577-648 | QQQQFPQQQFLQPQQQLSQQPEQIIPQQPQQQFPQQQFQPIPYPPQQQSQEPSPYQQYPQQQPSPLGSDVIS |
| >Consensus/LMW-GS/577-632 | QLAQGTFLQPHQIAQLEVMTSIALRTLPTMCSVNVPLYSTTTSVPFGVGTGVGAYL---------------- |
| >Consensus/HMW-GS/577-648 | QQGGQLGQGAQDQQPGQGQQGQQPGQRQQDQQPGQGQQPGQGQQGYYPTSPQQPGQGQQGQYPAQGQQSGQG |
| >Consensus/B-hordein/334-333 | ------------------------------------------------------------------------ |
| >Consensus/B1-hordein/302-301 | ------------------------------------------------------------------------ |
| >Consensus/B3-hordein/311-310 | ------------------------------------------------------------------------ |
| >Consensus/C-hordein/413-412 | ------------------------------------------------------------------------ |
| >Consensus/D-hordein/577-648 | TTVSPHQGQQTTVSPHQGQQTTVSPHPGQQTTVSPHQGQQTTVSPHPGQQTTVSPHQGQQTTVSPHQGQQTT |
| >Consensus/γ1-hordein/306-305 | ------------------------------------------------------------------------ |
| >Consensus/γ3-hordein/337-336 | ------------------------------------------------------------------------ |
| >Consensus/γ-secalin/541-540 | ------------------------------------------------------------------------ |
| >Consensus/ω-secalin/394-393 | ------------------------------------------------------------------------ |
| >Consensus/avenin/355-354 | ------------------------------------------------------------------------ |
|  |  |
|  | 649∙∙∙∙∙∙∙∙660∙∙∙∙∙∙∙∙∙672∙∙∙∙∙∙∙∙∙684∙∙∙∙∙∙∙∙∙696∙∙∙∙∙∙∙∙∙708∙∙∙∙∙∙∙720 |
|  | \|∙∙∙∙\|∙∙∙∙∙\|∙∙∙∙∙\|∙∙∙∙∙\|∙∙∙∙∙\|∙∙∙∙∙\|∙∙∙∙∙\|∙∙∙∙∙\|∙∙∙∙∙\|∙∙∙∙∙\|∙∙∙∙∙\|∙∙∙∙∙\| |
| >Consensus/α-gliadin/608-607 | ------------------------------------------------------------------------ |
| >Consensus/γ-gliadin/563-562 | ------------------------------------------------------------------------ |
| >Consensus/ω-gliadin/649-653 | ISGLE------------------------------------------------------------------- |
| >Consensus/LMW-GS/633-632 | ------------------------------------------------------------------------ |
| >Consensus/HMW-GS/649-720 | QPGYYPTSSQQPGQGQQPGQGQQGQQVQPGQGQQGQQPGQGQQPGQGQQGYYPTSPQQPGQGQQPGQGQPGY |
| >Consensus/B-hordein/334-333 | ------------------------------------------------------------------------ |
| >Consensus/B1-hordein/302-301 | ------------------------------------------------------------------------ |
| >Consensus/B3-hordein/311-310 | ------------------------------------------------------------------------ |
| >Consensus/C-hordein/413-412 | ------------------------------------------------------------------------ |
| >Consensus/D-hordein/649-720 | VSPHQGQQTTVSPHQGQQTTVSPHQGQQTTVSPHQGQQPGEQPCGFPGQQTTVSLHHGQQSNELYYGSPYHV |
| >Consensus/γ1-hordein/306-305 | ------------------------------------------------------------------------ |
| >Consensus/γ3-hordein/337-336 | ------------------------------------------------------------------------ |
| >Consensus/γ-secalin/541-540 | ------------------------------------------------------------------------ |
| >Consensus/ω-secalin/394-393 | ------------------------------------------------------------------------ |
| >Consensus/avenin/355-354 | ------------------------------------------------------------------------ |
|  |  |
|  | 721∙∙∙∙∙∙∙∙732∙∙∙∙∙∙∙∙∙744∙∙∙∙∙∙∙∙∙756∙∙∙∙∙∙∙∙∙768∙∙∙∙∙∙∙∙∙780∙∙∙∙∙∙∙792 |
|  | \|∙∙∙∙\|∙∙∙∙∙\|∙∙∙∙∙\|∙∙∙∙∙\|∙∙∙∙∙\|∙∙∙∙∙\|∙∙∙∙∙\|∙∙∙∙∙\|∙∙∙∙∙\|∙∙∙∙∙\|∙∙∙∙∙\|∙∙∙∙∙\| |
| >Consensus/α-gliadin/608-607 | ------------------------------------------------------------------------ |
| >Consensus/γ-gliadin/563-562 | ------------------------------------------------------------------------ |
| >Consensus/ω-gliadin/654-653 | ------------------------------------------------------------------------ |
| >Consensus/LMW-GS/633-632 | ------------------------------------------------------------------------ |
| >Consensus/HMW-GS/721-792 | YPTSPQQSGQGQQPGQLQQPAQGQKGQQPGQGQQGQQPGQGQQGQQPGQGQQGQQPGQGQQGYYPTSPQQSG |
| >Consensus/B-hordein/334-333 | ------------------------------------------------------------------------ |
| >Consensus/B1-hordein/302-301 | ------------------------------------------------------------------------ |
| >Consensus/B3-hordein/311-310 | ------------------------------------------------------------------------ |
| >Consensus/C-hordein/413-412 | ------------------------------------------------------------------------ |
| >Consensus/D-hordein/721-757 | SVEQPSASLKVAKAQQLAAQLPAMCRLEGGGGLLASQ----------------------------------- |
| >Consensus/γ1-hordein/306-305 | ------------------------------------------------------------------------ |
| >Consensus/γ3-hordein/337-336 | ------------------------------------------------------------------------ |
| >Consensus/γ-secalin/541-540 | ------------------------------------------------------------------------ |
| >Consensus/ω-secalin/394-393 | ------------------------------------------------------------------------ |
| >Consensus/avenin/355-354 | ------------------------------------------------------------------------ |
|  |  |
|  | 793∙∙∙∙∙∙∙∙804∙∙∙∙∙∙∙∙∙816∙∙∙∙∙∙∙∙∙828∙∙∙∙∙∙∙∙∙840∙∙∙∙∙∙∙∙∙852∙∙∙∙∙∙∙864 |
|  | \|∙∙∙∙\|∙∙∙∙∙\|∙∙∙∙∙\|∙∙∙∙∙\|∙∙∙∙∙\|∙∙∙∙∙\|∙∙∙∙∙\|∙∙∙∙∙\|∙∙∙∙∙\|∙∙∙∙∙\|∙∙∙∙∙\|∙∙∙∙∙\| |
| >Consensus/HMW-GS/793-864 | QGQQGHYPTSSQQPTQSQQPGQGQQGQQVGQGQQAQQPASQQQPGQGQQGYYPTSPQQPGQGQQSGQGQQGY |
|  |  |
|  | 865∙∙∙∙∙∙∙∙876∙∙∙∙∙∙∙∙∙888∙∙∙∙∙∙∙∙∙900∙∙∙∙∙∙∙∙∙912∙∙∙∙∙∙∙∙∙924∙∙∙∙∙∙∙936 |
|  | \|∙∙∙∙\|∙∙∙∙∙\|∙∙∙∙∙\|∙∙∙∙∙\|∙∙∙∙∙\|∙∙∙∙∙\|∙∙∙∙∙\|∙∙∙∙∙\|∙∙∙∙∙\|∙∙∙∙∙\|∙∙∙∙∙\|∙∙∙∙∙\| |
| >Consensus/HMW-GS/865-936 | YPTSPQQPGQGQQPGQGQQRQQPGQGQQTGQGQQGQQPAQVQQGQQPAQGQQGQQLGQGQQGQQPGQGQQGQ |
|  |  |
|  | 937∙∙∙∙∙∙∙∙948∙∙∙∙∙∙∙∙∙960∙∙∙∙∙∙∙∙∙972∙∙∙∙∙∙∙∙∙984∙∙∙∙∙∙∙∙∙996∙∙∙∙∙∙1008 |
|  | \|∙∙∙∙\|∙∙∙∙∙\|∙∙∙∙∙\|∙∙∙∙∙\|∙∙∙∙∙\|∙∙∙∙∙\|∙∙∙∙∙\|∙∙∙∙∙\|∙∙∙∙∙\|∙∙∙∙∙\|∙∙∙∙∙\|∙∙∙∙∙\| |
| >Consensus/HMW-GS/937-1008 | QGAQGQQPGQGQQGGQGGQGQQPGQGQSGYFPTSRQQSGQGQQPGQGQQGQQGQQSGQGQQGQQPGQGQQPG |
|  |  |
|  | 1009∙∙∙∙∙∙∙1020∙∙∙∙∙∙∙∙1032∙∙∙∙∙∙∙∙1044∙∙∙∙∙∙∙∙1056∙∙∙∙∙∙∙∙1068∙∙∙∙∙1080 |
|  | \|∙∙∙∙\|∙∙∙∙∙\|∙∙∙∙∙\|∙∙∙∙∙\|∙∙∙∙∙\|∙∙∙∙∙\|∙∙∙∙∙\|∙∙∙∙∙\|∙∙∙∙∙\|∙∙∙∙∙\|∙∙∙∙∙\|∙∙∙∙∙\| |
| >Consensus/HMW-GS/1009-1080 | QGQQGYYPTSPQQSGQGQQPGQWQQPGQGQPGYYPTSPQQQPGQGQQGMAASHYPASLQQPGQGQLGYYPTS |
|  |  |
|  | 1081∙∙∙∙∙∙∙1092∙∙∙∙∙∙∙∙1104∙∙∙∙∙∙∙∙1116∙∙∙∙∙∙∙∙1128∙∙∙∙∙∙∙∙1140∙∙∙∙∙1152 |
|  | \|∙∙∙∙\|∙∙∙∙∙\|∙∙∙∙∙\|∙∙∙∙∙\|∙∙∙∙∙\|∙∙∙∙∙\|∙∙∙∙∙\|∙∙∙∙∙\|∙∙∙∙∙\|∙∙∙∙∙\|∙∙∙∙∙\|∙∙∙∙∙\| |
| >Consensus/HMW-GS/1081-1152 | PQQPGQLQQPTQGQQPAQGQQGQRLAQGQQGQQPGQGQQGQQPAQGQQEEIKAILYGQQLGQGQQSQQEQQP |
|  |  |
|  | 1153∙∙∙∙∙∙∙1164∙∙∙∙∙∙∙∙1176∙∙∙∙∙∙∙∙1188∙∙∙∙∙∙∙∙1200∙∙∙∙∙∙∙∙1212∙∙∙∙∙1224 |
|  | \|∙∙∙∙\|∙∙∙∙∙\|∙∙∙∙∙\|∙∙∙∙∙\|∙∙∙∙∙\|∙∙∙∙∙\|∙∙∙∙∙\|∙∙∙∙∙\|∙∙∙∙∙\|∙∙∙∙∙\|∙∙∙∙∙\|∙∙∙∙∙\| |
| >Consensus/HMW-GS/1153-1224 | GQGQQPGQGQQGQQPGQGQQGQQPGQGQQPGQGQPASGTFLLNHSGYYPTSLMQSIALPEDPHMCKINRPKK |
|  |  |
|  | 1225∙∙∙∙∙∙∙1236∙∙∙∙∙∙∙∙1248∙∙∙∙∙∙∙∙1260∙∙∙∙∙∙∙∙1272∙∙∙∙∙∙∙∙1284∙∙∙∙∙1296 |
|  | \|∙∙∙∙\|∙∙∙∙∙\|∙∙∙∙∙\|∙∙∙∙∙\|∙∙∙∙∙\|∙∙∙∙∙\|∙∙∙∙∙\|∙∙∙∙∙\|∙∙∙∙∙\|∙∙∙∙∙\|∙∙∙∙∙\|∙∙∙∙∙\| |
| >Consensus/HMW-GS/1225-1296 | ETDISMPAGILGQGQPGYYPTSPQQSGQGQQPGQGQQPEQWQQPGQGQPGYYPTTSPQQPGQGQQGYYPTSP |
|  |  |
|  | 1297∙∙∙∙∙∙∙1308∙∙∙∙∙∙∙∙1320∙∙∙∙∙∙∙∙1332∙∙∙∙∙∙∙∙1344∙∙∙∙∙∙∙∙1356∙∙∙∙∙1368 |
|  | \|∙∙∙∙\|∙∙∙∙∙\|∙∙∙∙∙\|∙∙∙∙∙\|∙∙∙∙∙\|∙∙∙∙∙\|∙∙∙∙∙\|∙∙∙∙∙\|∙∙∙∙∙\|∙∙∙∙∙\|∙∙∙∙∙\|∙∙∙∙∙\| |
| >Consensus/HMW-GS/1297-1368 | QQPGQGQQPGQPEQPGQGQQPRQGQQGYYPISPQQPGQWQQSGQGQQGYYPTSPQQSGQGQQPGQWLQPGQW |
|  |  |
|  | 1369∙∙∙∙∙∙∙1380∙∙∙∙∙∙∙∙1392∙∙∙∙∙∙∙∙1404∙∙∙∙∙∙∙∙1416∙∙∙∙∙∙∙∙1428∙∙∙∙∙1440 |
|  | \|∙∙∙∙\|∙∙∙∙∙\|∙∙∙∙∙\|∙∙∙∙∙\|∙∙∙∙∙\|∙∙∙∙∙\|∙∙∙∙∙\|∙∙∙∙∙\|∙∙∙∙∙\|∙∙∙∙∙\|∙∙∙∙∙\|∙∙∙∙∙\| |
| >Consensus/HMW-GS/1369-1440 | LQSGYYLGATSPQQLGQGQQPGQWLQPGQGQQGYYPTSPQQSGQAQQPGQGQQIGQVQQPGQWLQPGQGQQG |
|  |  |
|  | 1441∙∙∙∙∙∙∙1452∙∙∙∙∙∙∙∙1464∙∙∙∙∙∙∙∙1476∙∙∙∙∙∙∙∙1488∙∙∙∙∙∙∙∙1500∙∙∙∙∙1512 |
|  | \|∙∙∙∙\|∙∙∙∙∙\|∙∙∙∙∙\|∙∙∙∙∙\|∙∙∙∙∙\|∙∙∙∙∙\|∙∙∙∙∙\|∙∙∙∙∙\|∙∙∙∙∙\|∙∙∙∙∙\|∙∙∙∙∙\|∙∙∙∙∙\| |
| >Consensus/HMW-GS/1441-1512 | YYPTSLQQSGQGQQSGQGQQGYYPTSGQQPGQGQQSGQGQQGYDSPHRTRATTRTRATRAATRTRATIRTRA |
|  |  |
|  | 1513∙∙∙∙∙∙∙1524∙∙∙∙∙∙∙∙1536∙∙∙∙∙∙∙∙1548∙∙∙∙∙∙∙∙1560∙∙∙∙∙∙∙∙1572∙∙∙∙∙1584 |
|  | \|∙∙∙∙\|∙∙∙∙∙\|∙∙∙∙∙\|∙∙∙∙∙\|∙∙∙∙∙\|∙∙∙∙∙\|∙∙∙∙∙\|∙∙∙∙∙\|∙∙∙∙∙\|∙∙∙∙∙\|∙∙∙∙∙\|∙∙∙∙∙\| |
| >Consensus/HMW-GS/1513-1584 | TRVLSNYHVSAEHQAASLKVAKARTRATRVLPNFSAAARQQLAAQLPAMCRLEGGDALSASQSFSAAARTRA |
|  |  |
|  | 1585∙∙∙∙∙∙∙1596∙∙∙∙∙∙∙∙1608∙∙∙∙∙∙∙∙1620∙∙∙∙∙∙∙∙1632∙∙∙∙∙∙∙∙1644∙∙∙∙∙1656 |
|  | \|∙∙∙∙\|∙∙∙∙∙\|∙∙∙∙∙\|∙∙∙∙∙\|∙∙∙∙∙\|∙∙∙∙∙\|∙∙∙∙∙\|∙∙∙∙∙\|∙∙∙∙∙\|∙∙∙∙∙\|∙∙∙∙∙\|∙∙∙∙∙\| |
| >Consensus/HMW-GS/1585-1589 | TRAAP------------------------------------------------------------------- |
