# Supplementary material for: Evaluation of the discriminatory potential of antibodies created from synthetic peptides derived from wheat, barley, rye and oat gluten
Source: PLoS One. 2021 Sep 23;16(9):e0257466. doi: 10.1371/journal.pone.0257466 (PMC8459967; doi:10.1371/journal.pone.0257466)
Supplement: Attachment — Submitted filename: Response to Reviewers.docx [file pone.0257466.s002.docx]

July 09^th^, 2021

**Prof. Patrizia Restani**

*PLOS one*

Università degli Studi di Milano

Dear Professor **Restani**,

We are pleased to submit our revised manuscript “Evaluation of the discriminatory potential of antibodies created from synthetic peptides derived from wheat, barley, rye and oat gluten” for consideration for publication in *PLOS one*. please acknowledge the following justifications for the raised points by the reviewers.

**Reviewer #1:**

***1) Apart from cereal flour, is the approach potentially applicable also to processed and final food products (heat-treated food, fermented food such as beer) ? This is an important point in order to evaluate the possible applicability of the approach for monitoring of “gluten-free” food products monitoring as far as gluten content quantification.***

It is expected that this approach will work for heated products. For fermented products, we cannot give a firm answer. In our opinion, it is too premature to talk about applicability for processed products as our work was not designed for this purpose. The object of the present manuscript was to validate a protocol for raising antibodies based on synthetic peptides in order to obtain specific antibodies.

As mentioned at lines 348 to 351 of the manuscript, further experimentations are planned with monoclonal antibodies. These experimentations will include the production of ELISA tests and a validation of the developed ELISA tests against multiple food matrices with incurred gluten. Clarifications have been made to line 349-350 of the manuscript.

***2) It is not clear from the discussion how this type of discrimination could also complement current immunoassays by settling the issue of over and underestimation of gluten content, thus improving the safety of food intended to CD and wheat-allergic patients, because it is not clear if this approach could target also gliadins in processed foods. Is it the test on denatured proteins sufficient to foreseen such a possible application?***

Over and underestimation of gluten in food products is the result of the way gluten is quantified (it is a side effect of the R5 test). The main factor for overestimation and underestimation is the origin of gluten. (Lines 38-40) The production of specific antibodies would resolve this issue because it will be possible to know the origin of gluten. Clarifications have been added in the text line 47-48.

As mentioned at lines 42 to 47 of the manuscript: “The use of a reference material for each grain, namely wheat, barley, rye and oats, as a calibration standard makes it possible to correct or at least reduce this variability”. However, “calibration is often impossible in food analysis, since the source of gluten, if present due to cross-contamination, is usually unknown”. Therefore, being able to identify the source of gluten in a food sample would help in selecting the appropriate calibration standards.

***3) LC-MS/MS have been already used to differentiate gluten sources. The possibility to set up an efficient complementary ELISA methodology is very interesting, but it is not clear from the presented results if this approach could be efficacious also on processed food obtained from flour or cereal ingredients.***

We agreed that LC-MS/MS is a way better tool than ELISA to differentiate gluten sources. However, LC-MS/MS are still marginal in food industries especially for the detection of gluten because the reference method is ELISA. The ultimate goal of this project is to develop tools that can be used by food industries, and considering their limited financial resources, the development of a new ELISA test seemed to be a good option. However, as mentioned at lines 53-54: “The aim of this study was to create new antibodies capable of distinguishing between different sources of gluten.”

ELISA tests need to be validated against several matrices before commercialization. We are currently in the process to produce these kits so talking about processed products is too early (see response to question 1).

**Reviewer #2:**

All “ml” unit notations have been changed to “mL”.

Fig 2 quality has been improved and colors have been added to the figure and legend for a better understanding.

Thank you for your consideration.

Warm regards,

David Poirier

*Université Laval*
